# Neural correlates of psychodynamic and non-psychodynamic therapies in different clinical populations through fMRI: A meta-analysis and systematic review

**DOI:** 10.3389/fnhum.2022.1029256

**Published:** 2022-12-20

**Authors:** Nicoletta Cera, Jessica Monteiro, Roberto Esposito, Giulia Di Francesco, Dietmar Cordes, Jessica Z. K. Caldwell, Filippo Cieri

**Affiliations:** ^1^Faculty of Psychology and Education Sciences, University of Porto, Porto, Portugal; ^2^CIBIT-Coimbra Institute for Biomedical Imaging and Translational Research, Coimbra, Portugal; ^3^Department of Radiology, Area Vasta 1/ASUR Marche, Pesaro, Italy; ^4^Hematology Unit, Pescara Hospital, Pescara, Italy; ^5^Department of Neurology, Cleveland Clinic Lou Ruvo Center for Brain Health, Las Vegas, NV, United States; ^6^Department of Brain Health, University of Nevada, Las Vegas, NV, United States; ^7^Department of Psychology and Neuroscience, University of Colorado, Boulder, CO, United States

**Keywords:** neural correlate, psychotherapy, psychoanalysis, meta-analysis, fMRI, psychodynamic neuroscience

## Abstract

**Background:**

The COVID-19 pandemic has exacerbated the ongoing crisis in psychiatric and psychological care, contributing to what we have identified as *a new psychological and psychiatric pandemic*. Psychotherapy is an effective method for easing the psychological suffering experienced also by the various impacts of COVID-19. This treatment can be examined from a neurological perspective, through the application of brain imaging techniques. Specifically, the meta-analysis of imaging studies can aid in expanding researchers' understanding of the many beneficial applications of psychotherapy.

**Objectives:**

We examined the functional brain changes accompanying different mental disorders with functional Magnetic Resonance Imaging (fMRI), through a meta-analysis, and systematic review in order to better understand the general neural mechanism involved in psychotherapy and the potential neural difference between psychodynamic and non-psychodynamic approaches.

**Data sources:**

The Preferred Reporting Items for Systematic Reviews and Meta-Analyses (PRISMA) guidelines were employed for our systematic review and meta-analysis. We conducted a computer-based literature search, following the Population, Intervention, Comparison and Outcomes (PICO) approach, to retrieve all published articles in English regarding the above-described topics from PubMed (MEDLINE), Scopus, and Web of Science.

**Study eligibility criteria, participants, and interventions:**

We combined terms related to psychotherapy and fMRI: (“psychotherapy” [All Fields] OR “psychotherapy” [MeSH Terms] OR “psychotherapy” [All Fields] OR “psychotherapies” [All Fields] OR “psychotherapy s” [All Fields]) AND (“magnetic resonance imaging” [MeSH Terms]) OR (“magnetic”[All Fields] AND “resonance”[All Fields] AND “imaging”[All Fields]) OR (“magnetic resonance imaging”[All Fields] OR “fmri”[All Fields]). We considered (1) whole brain fMRI studies; (2) studies in which participants have been involved in a clinical trial with psychotherapy sessions, with pre/post fMRI; (3) fMRI results presented in coordinate-based (x, y, and z) in MNI or Talairach space; (4) presence of neuropsychiatric patients. The exclusion criteria were: (1) systematic review or meta-analysis; (2) behavioral study; (3) single-case MRI or fMRI study; and (4) other imaging techniques (i.e., PET, SPECT) or EEG.

**Results:**

After duplicates removal and assessment of the content of each published study, we included 38 sources. The map including all studies that assessed longitudinal differences in brain activity showed two homogeneous clusters in the left inferior frontal gyrus, and caudally involving the anterior insular cortex (*p* < 0.0001, corr.). Similarly, studies that assessed psychotherapy-related longitudinal changes using emotional or cognitive tasks (TASK map) showed a left-sided homogeneity in the anterior insula (*p* < 0.000) extending to Broca's area of the inferior frontal gyrus (*p* < 0.0001) and the superior frontal gyrus (*p* < 0.0001). Studies that applied psychodynamic psychotherapy showed Family-Wise Error (FWE) cluster-corrected (*p* < 0.05) homogeneity values in the right superior and inferior frontal gyri, with a small cluster in the putamen. No FWE-corrected homogeneity foci were observed for Mindful- based and cognitive behavioral therapy psychotherapy. In both pre- and post-therapy results, studies showed two bilateral clusters in the dorsal anterior insulae (*p* = 0.00001 and *p* = 0.00003, respectively) and involvement of the medial superior frontal gyrus (*p* = 0.0002).

**Limitations:**

Subjective experiences, such as an individual's response to therapy, are intrinsically challenging to quantify as objective, factual realities. Brain changes observed both pre- and post-therapy could be related to other factors, not necessary to the specific treatment received. Therapeutic modalities and study designs are generally heterogeneous. Differences exist in sample characteristics, such as the specificity of the disorder and number and duration of sessions. Moreover, the sample size is relatively small, particularly due to the paucity of studies in this field and the little contribution of PDT.

**Conclusions and implications of key findings:**

All psychological interventions seem to influence the brain from a functional point of view, showing their efficacy from a neurological perspective. Frontal, prefrontal regions, insular cortex, superior and inferior frontal gyrus, and putamen seem involved in these neural changes, with the psychodynamic more linked to the latter three regions.

## Introduction

Mental disorders represent a significant public health concern, producing an enormous economic burden for society and great suffering for patients as well as their families and communities. After almost 3 years of the COVID-19 pandemic, the situation has worsened, creating increased urgency to strengthen mental health systems in most countries (COVID-19 Mental Disorders Collaborators, 2021).

Although the biopsychosocial model is shared by the World Health Organization's International Classification of Functioning (WHO ICF), the previous biomedical model of mental disorders is nowadays anachronistic but still pervasive, with supporters among clinicians and researchers, thereby threatening the understanding of highly complex phenomena, such as the human mental suffering. In addition to a more complex etiopathogenesis of mental disorders supported by the biopsychosocial model, compared to the biomedical model, a more complex approach to therapy also exists. Among the major criticisms of the biomedical model, especially related to mental disorders, its reductive view of etiopathogenesis and approach to therapy are the most important concerns.

Pre-COVID studies have shown that at least one person out of two (50% of the high-income countries' population) will be diagnosed with a psychiatric disorder in their life (Wittchen et al., 2011), most commonly anxiety and depressive disorders. Antidepressants are currently among the most frequently prescribed medications worldwide, being taken by more than 10% of the general population annually in high-income countries (Jorm et al., 2017; Furukawa et al., 2021). More patients every year are on longer-term anti-depressant treatment. In the US, even before the COVID-19 pandemic, we observed an increase of almost 30% of patients who had been using antidepressants for more than 5 years, from 13% in 1996, to 44% in 2015 (Jorm et al., 2017; Luo et al., 2020). Still in the US, we observed an increase of 21% in the number of antidepressant prescriptions in February and March 2020, the highest point since the declaration of the COVID-19 pandemic (America's State of Mind Report, 2020).

This paper is neither about the efficacy of pharmacotherapy, nor the efficacy of psychotherapy, or the comparison between psychotherapy and pharmacotherapy, but a brief introduction can be helpful to establish the urgency of this topic described by our current work and the necessity of studies able to explore the effects of psychological therapies, from a behavioral and neurological point of view, especially in a post-pandemic era, in which society is experiencing *a new psychological and psychiatric pandemic*.

## Pharmacotherapy and psychotherapy

Taking into consideration only the most widespread source of mental suffering, with its pharmacological treatment, according to the Food and Drug Administration (FDA; Turner et al., 2008), the efficacy of antidepressant drugs for depression is quite small, with an effect size of 0.26 for Fluoxetine and Sertraline, 0.24 Citalopram, 0.31 for Escitalopram, 0.30 for Duloxetine, and with an overall effect size of FDA approved antidepressant drugs between 1987 and 2004 of 0.31. Other more recent studies have shown that antidepressant medications achieve effect sizes of between 0.24 (tricyclics) and 0.31 in case of SSRIs (Kirsch et al., 2008). When Kirsch et al. (2008) have used published and unpublished Food and Drug Administration (FDA) registration trials, to assess antidepressant efficacy, they found that antidepressants were not clinically significant for mild, moderate, and severe depression, with a mean drug–placebo difference of only 1.80 points on the Hamilton Depression Rating Scale. Some studies suggest there are medications less effective than a placebo (clomipramine; Cipriani et al., 2018). A more recent study (Almohammed et al., 2022) has considered changes in the quality of life over 2 years in Americans with depression who took antidepressants (any type) vs. the changes reported by those with the same diagnosis who did not use antidepressants. The study found no significant difference in the quality of life of this population.

A recent review has revealed a lack of randomized double-blind placebo-controlled trials for anxiety disorders and few studies comparing novel treatments to existing anxiolytic agents, concluding that although some randomized controlled trials for novel agents exist, these trials have largely been negative (Garakani et al., 2020).

Furukawa et al. (2021) have recently published a systematic review and meta-analysis of randomized controlled trials (RCTs) in which adult patients with major depressive disorder were randomized to acute treatment with a psychotherapy (PSY), a protocolized antidepressant pharmacotherapy (PHA), their combination (COM), standard treatment in primary or secondary care, or pill placebo, and were then followed up through a maintenance phase. According to this authoritative paper, psychotherapy shows more effectiveness than pharmacotherapy, both if these treatments is continued into the maintenance phase (PSY → PSY vs. PHA → PHA: OR = 1.53, 95% CI: 1.00–2.35) and if they were followed by discretionary treatment (PSY → naturalistic vs. PHA → naturalistic: OR = 1.66, 95% CI: 1.13–2.44). The same applied to PSY when compared with standard therapy through the acute and maintenance phases [PSY → PSY vs. standard treatment in primary or secondary care (STD): OR = 1.76, 95% CI: 0.97–3.21; PSY → nat vs. STD: OR = 1.83, 95% CI: 1.20–2.78]. In other words, PSY (and combination of PSY and PHA) has more enduring effects than PHA. Therefore, guidelines on the treatment choice for depression may need to be updated accordingly (Furukawa et al., 2021).

Another recent systematic review (Wakefield et al., 2021) confirmed the efficacy of psychotherapy, showing large pre-post treatment effect sizes for depression [*d* = 0.87, 95% CI (0.78–0.96), *p* < 0.0001] and anxiety [*d* = 0.88, 95% CI (0.79–0.97), *p* < 0.0001]. In the comparison between cognitive behavioral (CBT) and psychodynamic psychotherapy (PDT) studies and meta-analysis have shown similar efficacy (Leichsenring and Steinert, 2017; Steinert et al., 2017), although some authors point out that in the case of PDT the effects last longer—and even increase—after the end of the treatment, with an effect size of between 0.78 and 1.46, even for diluted and truncated forms of psychoanalytic therapy (Shedler, 2010; Solms, 2018). A quasi-experimental comparison found psychoanalysis but not long-term PDT to be superior to CBT on measures of depression at 3-year follow-up (Huber et al., 2012). However, some authors have found a worse performance of PDT compared to CBT (Barber et al., 2021).

When we compare PHA and PDT, some studies fail to identify differential effects (Salminen et al., 2008; Barber et al., 2012; Bloch et al., 2012; Zilcha-Mano et al., 2014). On the other hand, a recent meta-review of 61 meta-analyses covering 21 psychiatric disorders containing 852 trials and 137,126 subjects yielded larger effect sizes for PDT (0.58; 95% CI: 0.42–0.76) than PHA (0.40; 95% CI: 0.28–0.52) studies (Huhn et al., 2014).

All these studies clearly show that psychotherapy (PDT, CBT, and other psychological approaches) works, usually more effectively than PHA alone, and it works by modifying patients' symptoms, thinking patterns, beliefs, attitudes, emotional states, and behaviors, in the most widespread mental disorders, showing a good effect size, in general and compared to pharmacotherapy. Based on these results, it should be a priority of the clinical and research community to understand not if PSY works, but how this form of treatment is able to act on a neurological and neuropsychological level. Without claiming to be exhaustive, the current study uses meta-analytic and systematic review approaches to explore functional brain changes through functional Magnetic Resonance Imaging (fMRI) among different mental disorders, trying to understand the general neural mechanism involved in psychotherapy and potential differences between changes associated with PDT and non-PDT.

## Neural effects

As Marek et al. (2009) have recently pointed out, most brain-wide association studies (typically based on a sample size of 25 subjects) have shown inadequacy. These authors have used a meta-analytic approach on three of the largest neuroimaging datasets currently available—with a total sample size of around 50,000 individuals, revealing how the usual brain wide association studies are not appropriate in capturing inter-individual differences in brain structure or function and complex cognitive or mental health phenotypes (Marek et al., 2009). Therefore, together with big data, a meta-analytic approach can enlarge the sample size, increase the statistical power, and give a more accurate idea about general brain changes. From a psychological (especially psychodynamic) point of view, we also note that subjectivity should be taken into consideration, given this form of therapy is highly individualized, with the *cure* passing through the therapist; thus, the subjectivity of the therapist, the patient, and their relationship, should be taken in consideration. However, since meta-analyses are not based on individuality, but on mean values, this search for objectivity has historically kept some psychoanalysts distant or skeptical toward research in general and neuroscientific research in particular.

If subjectivity is given more consideration by clinicians, therapists, and scientists involved in individual-differences level research, using a meta-analytic approach, a compromise between quantity (sample size) and quality (subjectivity) might be more reasonably reached. Such a compromise would meet the need to reinforce an already existing bridge between neuroscience and psychoanalysis, a process yearned for by psychoanalysts and neuroscientists alike over the last 20 years, and started by Freud more than a century ago (Kandel, 1999, 2016; Kaplan-Solms and Solms, 2000; Solms and Turnbull, 2002; Carhart-Harris and Friston, 2010; Boeker et al., 2013; de Greck et al., 2013; Scalabrini et al., 2018; Solms, 2018, 2021; Cieri and Esposito, 2019; Cieri et al., 2021; Northoff and Scalabrini, 2021; Rabeyron, 2021, 2022; Cieri, 2022).

Effects of psychological therapies, similarly to pharmacotherapy, are capable of visualization through brain imaging methods (Kandel, 1999). Meta-analytic approaches of psychotherapies can play a fundamental role understanding their neural, together with their psychological effects. This approach uses brain imaging methods to explore potential structural or functional effects of the talking cure on the brain, finding similar dysfunction in limbic structures, amygdala, hippocampus, frontal cortex, cingulate cortex, and basal ganglions (Sözeri-Varma and Karadaǧ, 2012).

Abbass et al. (2012) described a meta-analysis of brain imaging studies from 11 sources analyzing any form of PDT treatment. The sample was composed of 2 randomized controlled trials, 5 controlled trials and 4 case series. The patient's cohort was affected by depression (atypical and typical), borderline personality disorder, panic disorder and somatoform disorder, investigated by a variety of neuroimaging methods to examine regional metabolic activity and synaptic neurotransmission before and after treatment. These authors found a general normalization of synaptic or metabolic activity in limbic, mid-brain and prefrontal regions, occurring in association with improved clinical outcomes. Patterns of neural activity or neurophysiological infrastructure in regions of the dorsolateral prefrontal cortex (DLPFC), orbital frontal cortex (OFC), anterior cingulate cortex (ACC), and amygdala were found to vary between patients and healthy controls before the psychotherapy, while after treatment, the patterns seen in patients resembled those of the controls.

Messina et al. (2013) conducted a similar meta-analytic study including 16 sources, regardless of the specific psychotherapy approach used. Different diagnosis and methods were analyzed: depression, post-traumatic stress disorder, and panic disorder, investigated both with resting state and task-related activation. They have also considered phobic patients through exposure-related activation method. Anxiety and depression studies showed consistent results for changes in the dorsomedial prefrontal cortex (DMPFC) and in the posterior cingulate cortex/precuneus (PCC/Prc). Some changes were also described at the level of temporal lobes, both in anxious/depressed and phobic patients.

We can find analogous results in similar regions coming from the systematic review by Franklin et al. (2016). They analyzed brain changes, taking in consideration 10 neuroimaging studies associated with cognitive behavioral therapy (CBT) of depression. This specific form of psychological treatment was mostly correlated with changes in the ACC, PCC, VMPFC/OFC, and amygdala/hippocampus. As the authors suggest, CBT appeared to decrease the resting state activity in the dorsal ACC. Researchers involved in this study suggest that this form of treatment can develop an increased capacity for “top-down” emotion regulation, which is employed when skills taught in CBT are engaged.

Another systematic review was conducted by Gotink et al. (2016) taking into consideration both the structural and functional neuronal in stress-reducing effects of the 8-week Mindfulness Based Stress Reduction (MBSR) and Mindfulness Based Cognitive Therapy (MBCT) program. They considered 21 fMRI studies, showing that functional and structural changes in the prefrontal cortex (PFC), cingulate cortex, insula, and hippocampus are similar to changes described in studies on traditional meditation practice. In addition, MBSR led to changes in the amygdala consistent with improved emotion regulation.

Sankar et al. (2018) focused their meta-analytic investigation on psychotherapy of major depressive disorder (MDD), measuring neural function and metabolism using functional Magnetic Resonance Imaging (fMRI), Positron Emission Tomography (PET), Single-photon Emission Computerized Tomography (SPECT) and Magnetic Resonance Spectroscopy (MRS). A significant group by time effect was found in left rostral ACC, in which patients showed increased activity following psychotherapy while healthy controls showed a decrease at follow up. Longitudinal treatment effects revealed reduced left precentral cortical activity in MDD patients. Findings could be indicative of improvements in emotion responsivity that may be achieved following a psychological treatment, as suggested by the authors of this study.

A more recent study comes from Thorsen et al. (2018) who conducted a systematic review and performed a meta-analysis (Seed- based d-Mapping) of 25 whole-brain neuroimaging studies using fMRI or PET comparing brain activation of Obsessive Compulsive Disorder (OCD) patients and healthy controls during presentation of emotionally-valenced vs. neutral stimuli. OCD patients show increased emotional processing-related activation in limbic, frontal, and temporal regions, compared to healthy controls. We can observe also here similar results in similar regions described in the above mentioned studies. Particularly, patients showed increased activation in the bilateral amygdala, right putamen, OFC extending into the ACC and VMPFC, middle temporal, and left inferior occipital cortices during emotional processing.

## Aims of the study

**Figure 1:**
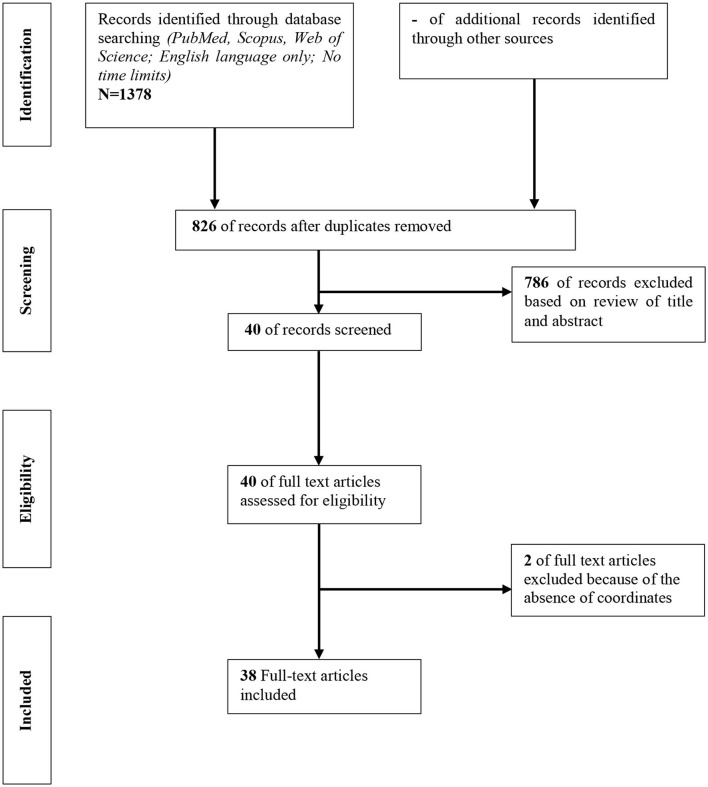
Flow diagram for identifying studies in the systematic review.

The objective of this meta-analysis and systematic review was to describe the neural correlates of psychological treatments. The distinctive element of our approach derives from the fact that we sought to systematically review the functional neural effects of psychotherapy, through the fMRI, both through the resting-state (rs-fMRI), and task-fMRI approaches, trying to limit the heterogeneity of imaging's tools used. We eliminated from our study structural methods (volumetric, cortical thickness, white matters etc.) and other brain imaging approaches (PET, SPECT, MRS, etc.). We considered an initial sample of 1,378 studies, reduced to 38 with a total sample size of 1,688 subjects (Figure 1). Another specificity of our approach is from a demographic and diagnostic point of views. We used the studies on adulthood, with an age range 18–65 years old, with two time points: before and after treatment; analyzing functional neural changes in major depressive disorder (MDD; 11 studies; 366 subjects), panic disorder (PD; 1 study; 27 subjects), somatoform disorder (SD; 2 studies; 120 subjects), social anxiety disorder (SAD; 5 studies; 169 subjects), generalized anxiety disorder (GAD; 2 studies; 57 subjects), post-traumatic stress disorder (PTSD; 7 studies; 412 subjects), obsessive compulsive disorder (OCD; 4 studies; 191 subjects), attention deficit hyperactivity disorder (ADHD; 1 study; 40 subjects), anhedonia (1 study; 73 subjects) and schizophrenia (SZ; 4 studies; 156 subjects). Moreover, from a psychotherapeutic perspective, we tried to outline as far as possible—conditioned by statistical limits deepened in the course of our work—the difference between psychodynamic (PDT; 4 studies) and non-PDT approaches (34 studies).

## Methods

The Preferred Reporting Items for Systematic Reviews and Meta-Analyses (PRISMA) guidelines has been used for our systematic review and meta-analysis (Moher et al., 2009).

In particular, our research question was specifically oriented toward functional neural changes related to psychotherapy intervention with different approaches (O) in psychologically healthy controls and psychiatric patients (P). The present review and meta-analysis was limited to longitudinal fMRI studies (I) with tasks and resting state (C). We defined the search terms based on the above mentioned PICO question combined with the Boolean operators “AND” and “OR”, according to the method previously used in other previously published systematic reviews (Cera et al., 2021; Vargas-Cáceres et al., 2021). We conducted a computer-based literature search follow the PICO approach combining terms related to psychotherapy and fMRI: (“psychotherapy” [All Fields] OR “psychotherapy” [MeSH Terms] OR “psychotherapy” [All Fields] OR “psychotherapies” [All Fields] OR “psychotherapy s” [All Fields]) AND (“magnetic resonance imaging” [MeSH Terms]) OR (“magnetic”[All Fields] AND “resonance”[All Fields] AND “imaging”[All Fields]) OR (“magnetic resonance imaging”[All Fields] OR “fmri”[All Fields]). We conducted the computer-based literature search to retrieve all the published articles in English regarding the above-described topics. We conducted our search in the three principal databases: PubMed (MEDLINE), Scopus and Web of Science.

Importantly, we identify fMRI studies on the basis of the following inclusion criteria: (1) whole brain fMRI studies; (2) Studies in which participants have been involved in a clinical trial with psychotherapy sessions; (3) fMRI results presented in coordinate-based (x, y, and z) in MNI or Talairach space; (4) presence of neuropsychiatric patients. The exclusion criteria were: (1) systematic review or meta-analysis; (2) behavioral study; (3) single-case MRI or fMRI study; (4) other imaging techniques (i.e., PET and SPECT) or EEG; (5) no coordinates.

The studies, which did not meet the above-mentioned criteria for the meta-analysis, will be included in our systematic review.

All of the included studies were screened to identify additional relevant bibliographic items. Similarly, the narrative, systematic reviews and meta-analyses were retrieved, and we screened them to find previous relevant articles in the reference lists.

After the duplicate removal, the title and abstracts were manually screened to determine if they fulfilled the inclusion and/or exclusion criteria. After the retrieval of potentially relevant studies, we read the full texts to confirm their eligibility.

Two authors conducted independently the literature search, screening, and methodological evaluation. The consensus about the different stages was reached between the two authors discussing the results and the articles retrieved. If a consensus was not reached, a third opinion was obtained.

To assess the quality of the studies included in the present systematic review, we applied the “NOS-scale” (Wells et al., 2022). Information has been extracted from each included study, following the above-mentioned guidelines. In particular, we extracted the characteristics of the participants, including the exclusion and inclusion criteria.

## ALE meta-analysis of the brain clusters resulting from the included fMRI studies

To assess the presence of a set of brain regions involved in the longitudinal above-mentioned studies, the brain coordinates reported in each included article were used for an ALE (activation likelihood estimation) meta-analysis. GingerAle 3.02 (Turkeltaub et al., 2012-https://www.brainmap.org/ale/) has been used to calculate the ALE meta-analysis. Indeed, GingerAle generates modeled maps of brain activations, by combining the probabilities of all brain activation foci for each voxel, as reported in the studies. Thus, the resulting maps are combined to obtain a voxel-wise ALE score. The ALE scores represent the convergence of results derived from the studies into a functional anatomical localization in the brain. The scores obtained are then compared with an empirical null distribution that represents a between—studies causal association (Eickhoff et al., 2012). Moreover, all coordinates following the stereotaxic space of the Montreal Neurological Institute-Hospital (MNI) have been transformed into the standardized 3D space of Talairach and Tournoux (1988) using Bioimage Suite (https://bioimagesuiteweb.github.io/webapp/mni2tal.html). To understand the role played by different brain regions in psychotherapy, we conducted three different ALE meta-analyses studies, with the cluster-level FWE correction (*p* < 0.01), where possible, or uncorrected statistical threshold with *p* < 0.005. According to Radua and Mataix-Cols (2012), a p threshold of 0.005 is reasonable. Moreover, to disentangle the contribution of the task-based and resting state studies, and to facilitate the interpretation of the results, a conjunction analysis has been performed using GingerALE, using a statistical threshold of *p* < 0.05 uncorrected. We used Mango 4.1 (http://ric.uthscsa.edu/mango/mango.html; Lancaster et al., 2010), which allows the visualization of results obtained by navigating between the volumes of the image of an MRI template in the Talairach stereotaxic space (1988) with 2 × 2 × 2 mm resolution (https://www.brainmap.org/ale/). Due to no contrast T2 > T1 (or interaction effect group x time) results reported in 2 studies, 36 studies have been included in the present meta-analysis.

## Results

**Figure 2:**
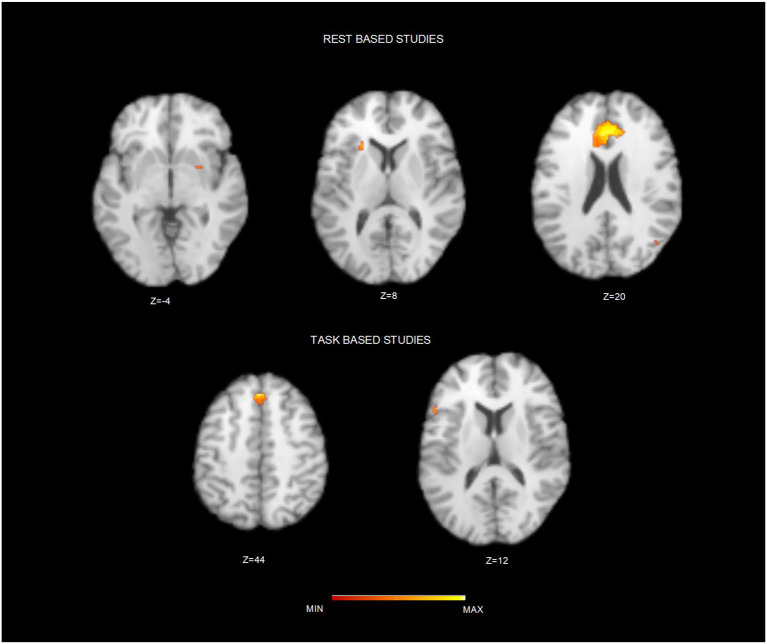
Brain clusters resulting from the conjunction analysis between resting state and task based studies. Figure depicts the resulting brain cluster after applying the conjunction analysis between resting state **(Top)** and task-based studies **(Down)**. Maps are over imposed on a Talairach template in the axial plane and in neurological convention.

After duplicates removal and the assessment of the content of each published study, we included 38 sources. Table 1 summarizes principal characteristics of the included studies in terms of demographics, the applied psychotherapy and a brief summary of the principal results as reported in each individual study. Furthermore, two authors have independently assessed the quality of the included studies applying the above-described NOS (New Castle–Ottawa Scale). All the studies included **1,688** participants with a range comprised between 18 and 65, and a median of 34.6 years old. Most of the studies (*n* = 28) applied cognitive-behavioral therapy (CBT) or non-psychodynamic, whereas specific PDT has been used in 4 studies, and specific mindfulness-based therapy in 6 sources. To assess the brain regions involved after a specific psychotherapy training, we conducted 3 different ALE meta-analyses (Table 2). The map including all the studies (ALL map) that assessed the longitudinal differences in brain activity, showed two homogeneous clusters in correspondence of left inferior frontal gyrus, concerning the orbitofrontal gyrus, and caudally involving the anterior portion of the insular cortex (*p* < 0.0001, corr.). Similarly, studies that assessed the longitudinal changes psychotherapy-related using emotional or cognitive tasks (TASK map) showed a left sided homogeneity in correspondence of the anterior insula (*p* < 0.000) extending to the Broca's area of the inferior frontal gyrus (*p* < 0.0001) and the superior frontal gyrus (*p* < 0.0001). Significant corrected results were not observed for the studies using resting state. After carrying out a conjunction analysis between resting state and task-based studies, for the resting state studies, uncorrected results (*p* < 0.006; *p* < 0.05) have been observed in correspondence of anterior cingulate cortex (ACC) and dorsal ACC (*p* < 0.006). Moreover, a small homogeneity cluster was found in the right Putamen (Table 3 and Figure 2). These results about resting state studies partially overlay the anatomofunctional localization of the one of the hubs of the default network (DN), located in vmPFC/ACC since the extracted coordinates were about FC results. Similarly, our interest was in studying the contribution of each type of psychotherapy to the brain functional response. Interestingly, the studies that used psychodynamic psychotherapy showed Family-Wise Error (FWE) cluster-corrected (*p* < 0.05) homogeneity values in correspondence of the right superior and inferior frontal gyri, with a small cluster in the putamen (Table 3).

**Table 1:** Characteristics of the included studies.

**References**	**Nr. Subjects (nr. of men)**	**Age (Mean years ±S.D.)**	**Diagnostic criteria**	**Rest vs. task**	**Therapy**	**MRI field**	**fMRI results**
Buchheim et al. (2012)	MDD: 16 (3); HC: 17 (4)	38.9 ± 12.4	DSM-IV	Task: Adult Attachment Projective Picture System used to activate AA system	PDT	3T	Interaction between Group × Time resulted in activation of left amygdala, hippocampus, STG, vACC, MPFC
Wiswede et al. (2014)	MDD: 18 (4); HC: 17 (3)	MDD: 39.8 ± 12.8 HC: 38.0 ±11.6	DSM-IV	Task: Operationalized Psychodynamic Diagnostics (OPD), traffic and relaxation sentences	PDT	3T	At T2 group × condition interaction, activation was observed in MFCG/ACC. No activation for OPD in MDD at T2.
Beutel et al. (2010)	PD: 9 (3); HC: 18 (9) 95	PD: 32 HC: 29	DSM-IV; ICD-10	Task: Emotional linguistic go/nogo task	PDT	1.5T	PD showed activation in SMA and lateral PFC for both emotional contexts. For positive Nogo > Positive go, at S2, PD showed increased activation in caudate nucleus.
Vuper et al. (2021)	PTSD: 26 ITT: 42 TEC: 18	PTSD: 33.62 ± 11.09 ITT: 31.17 ± 9.71 TEC: 31.89 ± 9.84	DSM-5	Rest ICA	CBT	3T	At T2 PTSD compared to TEC decreased connectivity between DMN and temporal and occipital regions. Changes in positive affectivity was related to connectivity between DMN, MTG and Lateral occipital cortex.
de Greck et al. (2013)	SD:15 (7) HC: 15 (7)	SD: 42.6 HC: 37.0	DSM-IV	Task: 1) Reward anticipation task; 2) empathy task.	PDT	1.5T	Bilateral hippocampus and left ITG showed augmented hemodynamic response. ROI-based results: For anger vs. control condition significant increase in post-central gyrus, STG, bilateral hippocampus, amygdala and insula
Dichter et al. (2009)	MDD: 12 (6) HC: 15 (6) 238	MDD: 39.0 ± 10.4 HC: 30.8 ± 9.6	DSM-IV	Task: The Wheel of Fortune (WOF)-decision-making task with selection, anticipation and feedback phases.	BATD	4T	Selection phase: Group × Time interaction showed increased activation in the MDD at T2 the paracingulate gyrus, the left putamen, the right supramarginal gyrus, and the left posterior temporal. Anticipation phase: MDD at T2: the left caudate, ACC, the left MFG and SFG, the left lingual gyrus, the left lateral and superior-lateral occipital cortex, the left Posterior parahippocampal gyrus, the right insula, right precuneus, right subcallosal cortex, right posterior temporal fusiform cortex, and bilateral precentral gyrus and temporal poles. Feedback phase: MDD at T2 were in the left planum temporale, right superior lateral occipital cortex, and right posterior temporal fusiform cortex.
Dichter et al. (2010)	DD: 12 (6) HC: 15 (6)	MDD: 39.0 ± 10.4SD HC: 30.8 ± 9.6	DSM-IV	Task: Sad vs. neutral images forced 2-choice task.	BATD	4T	Group × Time vs. Contrast Target Sad > Neutral showed increased activation at T2 in MDD in paracingulate gyrus
Fu et al. (2008)	MDD: 16 (3) HC: 16 (3)	MDD: 40.0 ± 9.4 HC: 39.20 ± 9.3	DSM-IV	Task: Sad faces morphed to represent 3 intensities. Subject had to indicate the gender of the faces	CBT	1.5T	ACC right MFG, right insula/IFG, and putamen showed a negative correlation with clinical response at T2. MDD showed a significant increase at T2 in : ACC, SFG, PCC/precuneus, inferior Parietal cortex.
Ritchey et al. (2011)	MDD: 11 (3) HC: 7 (2)	MDD: 36.1 ± 10.1SD HC: 34.6 ± 6.9	DSM-IV	Task: Emotion evaluation task (negative, neutral and positive stimuli) pleasantness assessment on a 3-point scale	CBT	1.5T	T1 differences between MDD and HC: reduction in vmPFC activity in MDD for the valence of the stimuli. T2: MDD increased activity of vmPFC.
Sankar et al. (2018)	MDD: 16 (3) HC: 16 (3)	MDD: 40 ± 9.27SD HC: 39.94 ± 9.48	DSM-IV	Task: mDAS-48 task (Dysfunctional Attitude Scale)	CBT	1.5T	MDD and HC showed a decrease in activation in the left parahippocampal gyrus at T2. In MDD, a main effect of time was observed in the right PCC. MDD: positive correlation between HAMD score and left precentral gyrus
Fonzo et al. (2017)	PTSD: 36 (13) HC: 30 (10) 413	PTSD: 34.42 ± 10.23SD HC: 39.03 ± 10.35	DSM-IV	Task : Emotion reactivity task; Emotional Conflict Task; Reappraisal Task	CBT; PE	3T	Reappraisal task: Increased activation in PTSD treatment group in MFG for condition decrease negative vs. look negative.
Fonzo et al. (2021)	PTSD: 36 (13) HC: 30 (10)	PTSD: 34.42 ± 10.23SD HC: 39.03 ± 10.35	DSM-IV	Rest Seed based connectivity	CBT; PE	3T	Significant treatment related increase in the amygdala connectivity with ventral anterior insula, DLPFC, vmPFC. Increased insula-amygdala connectivity at T2 of PE.
Huang et al. (2014)	MDD: 23 (7) HC: 20 (8)	MDD: 27.7 ± 10.9; HC: 28.8 ± 8.7	DSM-IV	Rest ReHo	CT	3T	At T1 MDD showed lower ReHo in the MPFC and rACC than HC. At T2 MDD showed no difference with HC.
Siegle et al. (2012)	MDD: 46 (9) HC: 35 (12) 4 cohort	Cohort 1 = 37.41 ± 9.17SD. Cohort 2 = 36.13 ± 11.11SD. Cohort 3 = 38.93 ± 9.74SD. Cohort 4 = 32.95 ± 9.37SD	DSM-IV	Task: Personal relevance rating task (PRRT).	CT	3T	Subgenual ACC activity was strongly related to changes and a more negative BDI in cohort 1.
Yoshimura et al. (2014)	MDD: 23 (16) HC: 15 (8)	37.3 ± 7.2	DSM-IV	Task: judgment tasks with self-reference, other-reference, semantic processing and letter-processing conditions.	CBT	1.5T	At T2 for group × time × valence, MDD dhowed increased activation for self/positive condition in vACC STG, MPFC. Similar results for self/negative codition. Increased activation for MDD between T1 and T2. Positive correlation between rumination scores and vACC at T2 for self/negative
Nakao et al. (2005)	OCD:10 (4) HC: 13 (6)	OCD: 32.4 ± 9.9SD; HC: 30 ± 6.4	DSM-III-R	Task: 1) Stroop task; 2) Symptom provocation task	BT; fluvoxamine	1.5T	Stroop task: At T2 OCD showed increased activation in bilateral prefrontal cortices, bilateral ACC, parietal cortices, and cerebellum. Symptom provocation task: At T2 OCD showed activation in OFC contrasting to Stroop task.
Bor et al. (2011)	SZ:8 (6) HC: 15 (10) 705	SZ: 30.5 ± 8.3; HC: 28.5 ± 7.2	DSM-IV-TR	Task: N-back, 0-back and 2-back	CRT	1.5T	At T2, SZ during the spatial WM showed activation within the left vlPFC the left ACC and left parietal areas.
Haut et al. (2010)	SZ: 18 (13) HC: 9 (7) 3 groups (REM, CBSST and Controls)	REM: 36.4 ± 9.2; CBSST: 39.5 ± 7.7; HC: 40 ± 6	DSM-IV	Task: 1) Word “N-back” 2) Picture “N-back” 3) Lexical decision Task.	REM; CBSST	3T	N-Back (word and picture): REM showed a number of regions with a significant activation > CBSST and HC: ACC, frontopolar, DLPFC. Lexical decision: no sig. results
Kumari et al. (2011)	SZ: 56 CBT + TAU group: 28 (18); TAU group: 28 (14)	SZ-CBT+TAU: 35.68 ± 7.82; SZ-TAU: 39.19 ± 9.37	DSM-IV	Task: Emotion recognition (sad, angry, happy and neutral faces)	CBT; Pharmacological	1.5T	Group × Time effect was found in bilateral IFG, right insula, bilateral putamen, left thalamus and left occipital areas (Fearful faces). CBT+ TAU showed decreased activation at aT2
Penadés et al. (2013)	SZ: 35 (27); HC: 15 (10)	SZ-CRT: 36.35 ± 13.6SD SZ- SST: 37.56 ± 8.99 HC: 34.75 ± 3.14	DSM-IV	Task: N-back task (0-back and 2-back)	CRT or SST	3T	Threated patients showed a reduction in overactivation of the CEN during task-related responses. Patients showed a reduction in deactivation of DMN. CRT group: increase in white matter in the genu of corpus callosum.
Nabeyama et al. (2008)	OCD: 11 (4) HC: 19 (8)	OCD: 32.4 ± 10.6; HC: 32.7 ± 7.1	DSM-III-R	Task: Stroop task.	E/RP	1.5T	At T2 OCD exhibited significantly decreased activation in the right OFC, left MFG, left fusiform gyrus, bilateral parahippocampal gyrus, and left parietal lobe; increased activation in the right parietal lobe
Goldin and Gross (2010)	SAD: 31 (NA) HC: 29(NA)	NA	DSM-IV	Task: Personally-salient autobiographical social situations	CBT	3T	Group × time allowed involvement of DLPFC and DMPFC in CBT group.
Ma et al. (2021)	OCD: 59 (22)	OCD-CCT: 27.2 ± 5.7 OCD-CCT-SSRI: 28.1 ± 5.1 OCD-SSRI: 28.1 ± 5.1	DSM-IV	Rest ALFF	CCT; pCCT/p	3T	No significant clusters (ALFF) at T2 in the 3 groups of OCD patients.
Zhu et al. (2018)	PTSD: 25 (7) HC: 26 (7)	PTSD: 35.3 ± 10.4SD; HC: 35.9 ± 8.9SD	DSM-IV-TR	Rest Seed based connectivity	PE	1.5T	Interaction group × time showed significant FC of the basolateral amygdala with OFC, vmPFC and thalamus.
Yoshino et al. (2018)	SD: 29 (9) HC: 31 (5)	SD: mean 46.7 ± 11.3; HC: 46.9 ± 10.3	DSM-IV	Rest ICA	Group CBT	3T	After CBT Intrinsic connectivity network (ICN) increased in OFC of DAN; decreased in right IPL of DAN and left PCL of SMN. At T2 negative correlation between VAS and right OFC. Vup
Sheynin et al. (2020)	PTSD: 61 (61) HC: 29 (13)	PT: = 35.23 ± S.D. = 8.6 HC = 32.38 S.D. = 8.27	DSM-IV- TR	Rest Seed based connectivity ROI-ROI connectivity	PE	3T	Statistical trend in the Amygdala-PCC connectivity for PTSD treatment responders.
Yuan et al. (2018)	SAD: 15 (10) HC: 19 (13)	SAD: 27.07 ± 8.11SD; HC: 26.6 ± 4.9SD	DSM-5	Rest ALFF	Group CBT	3T	Compared to T1, at T2 SAD showed decreased DC in left precuneus and MTG and increased in putamen. Positive correlation between DC in precuneus and LSAS.
Yuan et al. (2016)	SAD: 15 (10) HC: 19 (13)	SAD: 27.07 ± 8.11SD; HC: 26.6 ± 4.9SD	DSM-5	Rest Seed based connectivity	Group CBT	3T	Compared to T1, SAD abnormal hyperconnectivity between Amygadala and dACC, and improved clinical symptoms.
Pantazatos et al. (2020)	MDD:20 (12) HC: 30 (19)	MDD: 34.1 ± 10; HC: 32.4 ± 10	DSM-IV	Rest Seed based connectivity	CBT	3T	At T2, there was decreased connectivity in both rACC and BA25 and PCL, resulting in a more normalized pattern of activity.
Zhao et al. (2021)	OCD: 79 (32) 3 groups	Group 1: 30.8 ± 9.2SD. Group 2: 30.2 ± 9.4SD. Group 3: 29.1 ± 7.3SD.	DSM-IV	Rest Seed based connectivity	CCT; pharmacotherapy	3T	OCD under CCT treatment showed decreased FC of left Amygdala with right ACC and left precentral lobe.
Leroy et al. (2022)	PTSD: 30 (15) 2 groups	PTSD-responders: Mean = 41.3 ± 12.3SD PTSD-non responders: Mean = 36.6± 13.5SD	DSM-IV-TR	Rest FC assessed with Granger causality analysis	TMRT	3T	Significant changes in Anterior Insula causal maps after TMRT.
Scult et al. (2019)	GAD: 25 (9)	GAD: 21.8 ± 2.6	DSM-IV	Rest Seed based connectivity	ERT	3T	T2 > T1: 1) PCC seed: increased connectivity with middle occipital gyrus, precuneus cuneus, precentral gyrus/motor cortex and premotor areas/DLPFC. 2) Anterior insula seed: increased connectivity with precuneus. 3) Posterior insula seed: increased connectivity with anteromedial PFC/dACC and decreased connectivity with midbrain.
King et al. (2016)	14 PTSD: 14 (NA) PCGT: 9 (NA)	PTSD: 32.43 ± 7.54SD PCGT: 31.67 ± 10.14	DSM-IV	Rest: Seed based connectivity Task: Emotion regulation task	MBET	3T	At T2: Increased PCC connectivity with bilateral DLPFC and dACC and between left amygdala, left hippocampus and dACC.
Zhao et al. (2019)	GAD: 32 (24)	33.62 ± 7.71SD	DSM-IV	Rest ReHo	MBCT	3T	Decreased ReHo in the right middle temporal gyrus, bilateral insula, bilateral STP, right MCC and left hippocampus; Increased PCC connectivity with ACC.
Goldin et al. (2009)	SAD: 25 (16)	35.2 ± 11.9	DSM-IV	Task: SRP task	MBSR	3T	Positive SRP: decreased BOLD response in dorsomedial e medial PFC, left inferior frontal gyrus. Negative SRP: increased BOLD response
Cernasov et al. (2021)	Anhedonia Subject: 73 (21) 2 groups (BATA and MBCT)	MBCT: 31.8 ± 9.2SD BATA: 27.9 ± 8.8	DSM-5	Rest ROI-to-ROI network connectivity	BATA and MBCT	7T	No significant between group differences.
Bachmann et al. (2018)	ADHD: 40 (NA) 2 groups (MAP and PE)	MAP: 40 ± 10.58SD; PE: 40.26 ± 13.81	DSM-IV	Task: Working memory task	MAP or PE	3T	MAP: activations in bilateral inferior parietal lobule, posterior insula and precuneus.
Goldin and Gross (2010)	SAD: 16 (7)	35.2 ± 11.9	DSM-IV	Task: Emotion regulation task	MBSR	3T	Activations in inferior and superior parietal lobule, precuneus, cuneus, middle occipital gyrus and parahippocampal gyrus.

HC, Healthy control; Dx, Diagnosis; MDD, Major Depression Disorder; PD, Panic Disorder; PTSD, Post-Traumatic Stress Disorder; SZ, Schizophrenia; SAD, Social Anxiety Disorder; SD, Somatoform Disorder; GAD, General Anxiety Disorder; OCD, Obsessive Compulsive Disorder; Anhe, Anhedonia; PDT, Psychodynamic Therapy; CBT, Cognitive Behavioral Therapy; BATD, Behavioral Activation Therapy for Depression; EP, Exposure Therapy; WL, Waiting List (not healthy; for instance Fonzo et al., 2021); BT, Behavioral therapy; CRT, Cognitive remediation therapy; CBSST, cognitive behavioral social skills training; REM, remediation training; TAU, Treatment as usual; SST, social skills training; MT, Mindfulness training; MBSR, Mindfulness-based stress reduction; E/RP, exposure and response prevention; NSBs, reappraising self-generated negative self-beliefs; MBET, mindfulness-based exposure therapy; MBCT, Mindfulness-based cognitive therapy; MBM, Mindfulness-based meditation; HEP, Health enhancement program; SRP, Self Referential Processing; MAP, Mindfulness Awareness Practice; CCT, Coping Cognitive Therapy; pCCT, pharmacological CCT; p, Pharmacological therapy; PE, Prolonged Exposure Therapy; BATA, Behavioral Activation Therapy for Anhedonia; TMRT, traumatic memory reactivation therapy; ERT, Emotion regulation therapy; ICA, Independent Component Analysis; ReHo, Region Homogeneity; ROI, Region of Interest; FC, Functional Connectivity; ALFF, Analysis Low Frequency Fluctuation.

**Table 2:** Brain clusters resulting from ALE meta-analysis performed for all psychotherapy, CBT, PDT, and mindfulness based studies.

**Cluster**	**BA**	**Hemisphere**	***x***	***y***	***z***	**ALE**	***P***	***Z***
**All psychotherapy studies**
Inferior frontal gyrus/anterior insula	13	L	−44	26	8	0.021	0.000009	4,285
Inferior frontal gyrus/ orbital gyrus	47	L	−42	22	2	0.020	0.000012	4,232
**CBT**
Anterior insula	13	L	−44	26	8	0.01977	0.00001	4.41716
Anterior insula	13	R	38	18	4	0.01728	0.00003	4.04779
Superior frontal gyrus	8	L	0	28	48	0.01783	0.00002	4.13672
**Psychodynamic psychotherapy**
Inferior frontal gyrus	47	R	21	21	−12	0.008569	0.000093	3.736320
Lentiform nucleus/putamen		L	−25	−12	7	0.008569	0.000093	3.736320
Superior frontal gyrus	8	R	3	44	49	0.008706	0.000053	3.875660
Anterior cingulate cortex	32	R	1	46	4	0.009100	0.000012	4.215530
**Mindfulness**
Inferior frontal gyrus	47	L	−44	22	0	0.01458	0.00000	4.55147

BA, Broadman Area; L, Left; R, Right.

**Table 3:** Brain clusters resulting from the conjunction analysis between resting state and task based studies.

**Cluster**	**BA**	**Hemisphere**	***x***	***y***	***z***	***p***	***Z***
**Conjunction analysis REST vs. TASK**							
dACC	32	R	4	32	22	0.0054	2,549
ACC	24	L	−2	30	22	0.0056	2,536
ACC	24	L	−1	32	15	0.0102	2,319
ACC	24	L	−8	32	18	0.0126	2,238
dACC	32	L	−12	22	18	0.0142	2,192
ACC	24	L	−4	18	22	0.0182	2,092
ACC	24	L	−8	24	14	0.0192	2,071
ACC	24	L	−10	18	20	0.027	1,927
Medial frontal gyrus	11	R	9	28	−12	0.0478	1,667
Putamen	–	R	24	0	−4	0.0384	1,770
Middle temporal gyrus/angular gyrus	39	R	48	−68	18	0.0432	1,715
Middle temporal gyrus/angular gyrus	39	R	46	−68	20	0.0474	1,671
Middle temporal gyrus/angular gyrus	39	R	44	−66	20	0.0474	1,671

BA, Broadmann Area; L, Left; R, Right; dACC, Dorsal anterior cingulate cortex; ACC, anterior cingulate cortex.

No FWE-corrected homogeneity foci have been observed for Mindful- based and CBT psychotherapy. Comparing T1 and T2, CBT studies showed two bilateral clusters in the dorsal anterior insulae (*p* < 0.001) and the involvement of the medial superior frontal gyrus (*p* < 0.0005). Moreover, after Mindful-based therapy, left inferior frontal gyrus showed uncorrected homogeneity (*p* < 0.0001).

**Figure 3:**
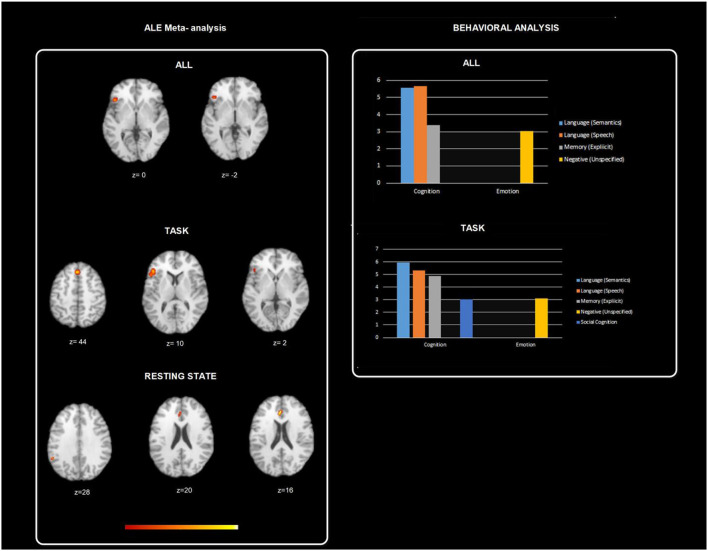
Figure depicts behavioral analysis of the ALE resulting maps using Behavioral Analysis plugin of Mango v.2.6. Behavioral Analysis presented for BrainMap's five Behavioral Domains (Action, Cognition, Emotion, Interoception, and Perception) and sixty sub-domains. Only *z*-scores ≥ 3.0 are considered significant (*p* ≤ 0.05 with Bonferroni corr.). All psychotherapies resulted in a map that allowed significant behavioral analysis for the domain cognition (Language and Memory; *z* = 5.09) and emotion (Negative; *z* = 3.04). ALE task map did not show significant results for the above-mentioned domains. ALE resting state fMRI map showed significant results for the domain emotion (Positive reward /Gain; *z* = 3.236). The observed homogeneous clusters in the CBT-ALE map allowed significant cognition domain (reasoning, language, memory, and attention) and emotion domain (reward).

Furthermore, Behavioral Analysis (Lancaster et al., 2012) of the ALE resulting maps has been performed using Behavioral Analysis plugin of Mango v.2.6 (http://ric.uthscsa.edu/mango/plugin_behavioralanalysis.html). Behavioral Analysis presented for BrainMap's five Behavioral Domains (Action, Cognition, Emotion, Interoception, and Perception) and sixty sub-domains. Only z-scores ≥ 3.0 are considered significant (*p* ≤ 0.05 with Bonferroni corr., Figure 3). All psychotherapies resulted in a map that allowed significant Behavioral analysis for the domain cognition (Language and Memory; *z* = 5.09) and emotion (Negative; *z* = 3.04). ALE task map did not show significant results for the above-mentioned domains. ALE resting state fMRI map showed significant results for the domain emotion (Positive reward /Gain; *z* = 3.236). The observed homogeneous clusters in the CBT-ALE map allowed significant cognition domain (reasoning, language, memory, and attention) and emotion domain (reward).

## Discussion

In the present study we sought to delineate the neural effects of shared and unique effects of different psychological therapies, evaluating the findings of existing longitudinal prospective studies in different psychopathological conditions. In other words, we tried to answer the question of how brain function changes after psychotherapy, using fMRI. Our second, related question was to assess the contribution of psychodynamic therapy from a neural point of view, underscoring any potential differences with non-PDT.

Our meta-analysis consisted of studies that had acquired serial neuroimaging scans prior to and following a course of treatment with a psychological therapy. Longitudinal brain imaging studies of both resting state and of emotional-cognitive tasks, using fMRI, were included, and analyzed separately and conjunctly.

## All studies together

The map including all studies that assessed longitudinal brain changes showed two homogeneous clusters from the orbitofrontal cortex (OFC), and caudally involving the anterior portion of the insular cortex. This finding is not surprising, both because of previous similar results existing in the literature and the specific functions of these regions. Specifically, Fu et al. (2013) found the OFC is one of the regions predictive of a good clinical response to psychotherapy. The prefrontal cortex, in general, has a complex role in the control and organization of behavior. In this context, the OFC is a fundamental part of the mesolimbic system, uniquely placed to integrate sensory and autonomic information to modulate behavior through both visceral and motor systems (Kringelbach, 2005). This structure is specialized in processing the reward value of numerous types of stimuli (Rolls et al., 2020). Greater activity in this area may reflect an increased responsivity to hedonic stimuli and reward, the inverse of anhedonia, which is predictive of a better clinical response (Fu et al., 2013). The OFC also has a role to connect and “make sense” of reality through sensory integration, modulation of visceral reactions, participating in learning, prediction and decision making for emotional and reward-related behaviors (Kringelbach, 2005). All these complex activities are critical for psychotherapy, from the embodied mind to emotion, learning, and prediction. Decreased functional connectivity of the OFC with medial temporal lobe areas involved in memory is found in patients with depression. Rolls et al. (2020) point out some therapies for depression could increase the activity or connectivity of the medial OFC.

The result at the insular cortex is even less surprising since this region conveys sensory information, in contact with the external reality, to further brain regions that allow processing. Insula is anatomically situated in a brain area connected with several functional neural circuits supporting cognitive, homeostatic, and affective systems. Its position and function represent a bridge between brain regions involved in monitoring internal states (visceral sensory, somatic sensory processes, autonomic regulation of the gastrointestinal tract and heart; Singer et al., 2004; Menon and Uddin, 2010; Esposito et al., 2018), supporting their processing. This pathway is called the “homeostatic afferent pathway” (Craig, 2009) carrying information about the body. Particularly, information arising from the body reaches the middle and posterior parts of the insula and then is projected in the anterior insula. The awareness of salient events is represented in the anterior insula, whereas more sensory attributes are represented posteriorly (Craig, 2002, 2003, 2009). Insula is a core structure that receives bodily information, filters salient stimuli, processes them, and then engages—through anterior cingulate cortex (ACC), the central executive network, and the default network (DN)—memory and functions connected to the self (Cieri and Esposito, 2019; Cieri, 2022). Given its functions, the insula is considered a central hub for the allostatic-homeostatic regulatory process between mother and infant (Scalabrini et al., 2022). This element makes considerable sense in a psychodynamic context, where the transferal phenomena between the analyst and the analysand can recall the mother and the infant. This region is also considered a bridge that connect the three level of self as recently proposed by Northoff and colleagues (Scalabrini et al., 2018, 2022; Qin et al., 2020).

## Emotional and cognitive tasks

The result at the anterior insula is also present in emotional and cognitive task studies, together with the inferior and superior frontal gyrus. Within the frontal lobe, inferior and superior frontal gyrus are the target influenced especially by PDT and mindfulness, while CBT was linked to significant modification of medial superior frontal gyrus. The inferior frontal gyrus corresponds to Broca's area, therefore involved in language processing, speech production, lexical and semantic processes, syntactic, and phonological processes, all functions present and vital in the context of the talking cure. Moreover, other fMRI research has shown that the blood oxygenation level dependent (BOLD) signal within the inferior frontal gyrus increases at the point of inhibitory control when compared to a baseline of routine responding (Menon et al., 2001). In other words, this area seems to play a key role in the inhibition processes (Verbruggen and Logan, 2008). This result makes sense in the task paradigm, and it also finds a natural expression within a psychotherapeutic treatment where the inhibition processes are often faced and discussed within treatment.

The superior frontal gyrus is thought to contribute to higher cognitive functions and particularly to working memory, but its activation during conflict anticipation is positively correlated with the capacity of inhibitory control associated with both efficient response inhibition and less motor urgency (Hu et al., 2016). Again, we find an involvement of inhibitory control through top-down processes or more focus on bottom-up mental states. We will take up these results about the superior and inferior frontal gyrus later in the discussion of the PDT.

## Resting state studies

Although significant corrected results were not observed for the studies using resting state, we will briefly discuss the uncorrected results at the level of another important area: the ACC. As we pointed out in a prior study (Cera et al., 2019) this structure is involved along with the insular cortex, the secondary somatosensory cortex, the nuclei in the tegmentum and the hypothalamus, in the regulation of attentional focus by integrating external and internal stimuli, and in the expression of emotional states, thus modulating a motivational state toward homeostasis (Damasio et al., 2000; Cera et al., 2019). Sankar et al. (2018), in their meta-analysis showed significant interaction effect of CBT to the rostral ACC in depressed patients compared to healthy controls. The findings could provide some insight into the potential mechanisms and specificity of treatment effects of this therapy. Patients with major depression showed increased activity following psychological therapy while healthy subjects have shown decreased activity at the follow up scan. In our current result, we found the involvement of the dorsal ACC, part of the salience network, implicated with emotional processing and the supramarginal gyrus, part of the DN, which plays a role in the therapeutic process (Cieri, 2022; Rabeyron, 2022). The poor specificity of our study (disorders and therapies) and the relatively small sample size could be among the reasons why the ACC did not survive multiple comparisons correction.

## Psychodynamic specificity

We also were interested in addressing the specific contribution of PDT. While the low number of psychodynamic studies in this field limits statistical power, the studies that used PDT showed Family-Wise Error (FWE) cluster-corrected (*p* < 0.05) homogeneity values in the right superior and inferior frontal gyri, with a small cluster in the putamen. We have already mentioned the potential role of the inferior and superior frontal gyri, extending beyond the important function of language to the potential inhibitory role stimulated or developed in therapy. We also note that the inferior frontal gyrus, as part of the frontal cortex, is a neocortical region that coordinate a wide range of neuropsychological processes (Miller and Cohen, 2001), with an important role modulating bottom-up process such as regulation of behavior that is more automatic, but also included in processes when behavior must be guided by internal states, with a more classic top-down function. Many regions in this area overlap with the human mirror neuron network (inferior frontal gyrus and superior temporal gyrus) involved in action observation and execution (Rizzolatti and Craighero, 2004). Modulation, inhibition processes, focus on internal states and the involvement of the mirror neuron network are vital components of any psychotherapy, in fact in this case a result present in PDT, CBT and mindfulness. The insula also has been shown to act in concert with the human mirror neuron system during imitation and observation of emotions (e.g., empathy; Carr et al., 2003; Uddin et al., 2009), which again, are expected to have a fundamental role in psychotherapy, not only as a requirement of the therapist and the patient within the therapy, but as a function potentially developed during treatment.

Concerning the insula, it is worth mentioning that the anterior portion of this region (and the von Economo neurons it contains) has a crucial role in awareness, and thus it needs to be considered as a potential neural correlate of consciousness (Craig, 2009) with a peculiar meaning for our study, and with a potential fundamental role in psychotherapy.

A specific region significant for the psychodynamic approach is the putamen. It is part of basal ganglia, which has long been assumed not only to play a role in motor planning and control, but also involved in several language aspects (Viñas-Guasch and Wu, 2017) including lexical, morphological (Friederici, 2002), syntactic (Teichmann et al., 2015), and speech production processes (Oberhuber et al., 2013). Moreover, the basal ganglia are involved in mammalian learning and memory (Aosaki et al., 1994; for a review see Packard and Knowlton, 2002). It should be noted that there is also extensive research examining the role of the basal ganglia in adaptation in motor control (Graybiel et al., 1994), neural representations of habits (Jog et al., 1999), space and direction and navigation (Wiener, 1993; Mizumori et al., 2000), explored also through neural computational modeling (e.g., Gillies and Arbuthnott, 2000). We can speculate that the involvement of this region in PDT—with a role not only on movement and language, but also involved in habits, neural representation, navigation, and memory—could be stimulated by a technique that works more with space-time, memory and dreaming, compared to more cognitive psychotherapy approaches that are focused on symptoms and the here and now, with less focus to the past and future (Cieri, 2022), landmark of psychoanalysis and PDT. We also found it intriguing that some authors (Bartels and Zeki, 2004; Zeki and Romaya, 2008) found equal activation of the network involving this region by love and hate (Zeki and Romaya, 2008). We can speculate that this overlapping has several theoretical and clinical psychodynamic precedents. In “Instinct and Their Vicissitudes”, Freud (1915) claims that love and hate characterize the natural relationships of the ego with the objects. Klein (1937) at the very beginning of her “Love, Guilt, and Reparation” points out how destructive impulses play in interaction of hate and love and how feelings of love and tendencies to reparation develop in connection with aggressive impulses and in spite of them. In fact, the baby's first object of love and hate—her mother—is both desired and hated with all the intensity and strength that is characteristic of the early urges of the baby. From a more clinical perspective, Winnicott (1949) in “Hate in the Counter-Transference”, resume Freud and Klein's perspectives, underscoring that these two affects overlap both in the patient, and the therapist. He points out that during the analysis of patients with and without psychosis, therapists must find themselves in a position comparable to that of the mother of a new-born baby, highlighting the presence of love and hate, both in the mother toward the baby and in the therapist toward the patient.

Before the conclusion and specifically with regard to the psychodynamic contribution, we want to use a metaphor from Kant (1781, 2003), from “Critique of Pure Reason” where he uses the metaphor of the dove to express something potentially similar to the dialogue between neuroscience and psychoanalysis:

“The light dove, in free flight cutting through the air the resistance of which it feels, could get the idea that it could do even better in airless space. Likewise, Plato abandoned the world of the senses because it posed so many hindrances for the understanding and dared to go beyond it on the wings of the ideas, in the empty space of pure understanding.”

This image of dove and its flight could be taken in consideration in this field thinking about some psychoanalysis that believes that it doesn't need methodological research, especially in the field of the brain sciences. On the other hand, neuroscientific research nowadays relies almost exclusively on algorithms and artificial intelligence without taking into consideration the subjective experience of patients. Today, a psychodynamic approach without dialogue with neuroscience sounds “brainless.” In contrast, brain neuroscientific research sounds “mindless” without the consideration of subjective individual experience (Cieri, 2022). Each approach needs the other side of the mind-brain system; according to Kant's metaphor, both sides need the resistance of the air to fly.

## Conclusions

In this work we systematically reviewed the evidence of effect of psychological therapy on brain function, trying to better understand the neurobiological bases of the effectiveness of psychotherapeutic treatments. All the psychological approaches seem to influence the brain from a functional point of view, showing their efficacy from a neurological perspective. It is not easy to delineate a precise and distinguished pattern of changes in specific disorders or theoretical approaches. Frontal, prefrontal regions, insular cortex, superior and inferior frontal gyri, and putamen seem involved in these neural changes, with the PDT more involved in the latest three regions.

## Limitations

Our study has limitations, such as the subjectivity of the individuals as response to the therapy. This element, of course is not a limitation *per se*, it is impossible to capture in its complexity, and it can be applied to the efficacy of the therapy in general. As Fonagy (2015) considered, it might be understandable to wish for an intellectual short-cut to a pooled effect size rather than considering individual investigations, but meta-analyses lack individual patient data—they are based on response rates and mean values. Being aware of this aspect, in our study we tried to discuss neural changes due to a relationship between two individuals (as a psychotherapeutic setting). Even before the limitation derived from putting together different therapeutic approaches, there is an intrinsic and ineliminable limitation in the objectification of a relationship unique par excellence. Related to this limitation, the pre-post therapy observed brain changes could be related to some other factors, not necessary to the specific treatment received. As underlined by other colleagues (Messina et al., 2013; Franklin et al., 2016) such studies are characterized by heterogeneity of therapeutic approaches and study designs. We have differences in sample characteristics, such as the specificity of the disorder (e.g., proportion experiencing first depressive episode vs. recurrent illness), number and duration of sessions, regions chosen for reduced threshold analyses, scanner resolution and nature/ existence of comparator group etc. that can be seen as potential confounders and limitations. As mentioned, some of these limitations are intrinsic and unavoidable. Another important limitation is the great variety of statistical analysis techniques used in previous resting state fMRI studies that we included. Since the methods to assess low frequency fluctuation, and their variation related to psychotherapy, of BOLD signal can be considered a bias in the study results. Indeed, we included several resting state studies that applied a seed-based connectivity analysis but using different seeds (Wolters et al., 2019). In this way, more studies applying the same analytical methods should be needed, allowing a more homogeneous and unbiased results as reported by previous meta-analysis (Iwabuchi et al., 2015). Finally, our sample size of the total studies used, is relatively small, particularly derived from the PDT little contribution. Specifically, as recalled by Eickhoff et al. (2016), cluster-level thresholding does a very good job of controlling excessive contribution of one experiment if 17 or more experiments are included in an ALE analysis. In our case, we have used 18 studies, but only 4 studies from PDT.

## Data availability statement

The original contributions presented in the study are included in the article/supplementary material, further inquiries can be directed to the corresponding author/s.

## Author contributions

NC and JM: investigation, methodology, formal analysis, visualization, and writing review and editing. RE, GD, DC, and JC: visualization and writing review and editing. FC: conceptualization, investigation, methodology, visualization, writing original draft, and writing review and editing. All authors contributed to the article and approved the submitted version.
